# Occupational Exposure Incidents Among Nursing Students: Knowledge, Experience, and Reporting Practices—A Cross-Sectional Study

**DOI:** 10.3390/nursrep16050166

**Published:** 2026-05-14

**Authors:** Mario Marendić, Ajka Pribisalić, Ivana Bokan, Ivana Parčina, Silvija Vladislavić, Mario Podrug, Ante Buljubašić, Anamarija Jurčev Savičević

**Affiliations:** 1 Faculty of Health Sciences, University of Split, 21000 Split, Croatia; mmarendic@fzz.unist.hr (M.M.); ivana.bokan@skole.hr (I.B.); ivana.parcina1@kbsplit.hr (I.P.); svladislav@fzz.unist.hr (S.V.); mpodrug@fzz.unist.hr (M.P.); abuljubasic@fzz.unist.hr (A.B.); anamarija.jurcev.savicevic@nzjz-split.hr (A.J.S.); 2 Health School Split, 21000 Split, Croatia; 3 University Hospital Center Split, 21000 Split, Croatia; 4 Department of Public Health, University of Split School of Medicine, 21000 Split, Croatia; 5 Teaching Institute of Public Health of Split-Dalmatia County, 21000 Split, Croatia

**Keywords:** occupational exposure, needlestick injury, students, nursing, accidents, occupational, infection control, health knowledge, attitudes, practice

## Abstract

**Background**: Nursing students are at high risk of exposure to blood and body fluids due to limited clinical experience. Ensuring adequate knowledge and proper post-exposure protocols is vital for improving safety and post-exposure management. **Aim**: This study aimed to evaluate the level of knowledge, previous exposure experience, and reporting practices regarding occupational exposure incidents among nursing students at the Faculty of Health Sciences, University of Split, Croatia. **Methods**: A cross-sectional study was conducted among 274 nursing students using a structured self-administered questionnaire. Descriptive statistical methods were applied, along with univariate and multivariate linear regression analyses. **Results**: Exposure incidents were experienced by 36.3% of students, with needlestick injuries being the most common (80.1%). In terms of reporting practices, fewer than half (40.8%) of those affected officially reported the incident. While students demonstrated adequate overall performance on the knowledge assessment (median score 12, IQR: 11–14), significant gaps were identified in hepatitis B and C protocols and immediate wound care. Multivariate analysis identified full-time student status (β = 1.24; *p* = 0.010) and first-year students (β = 0.82; *p* = 0.036) as factors significantly associated with higher knowledge scores. **Conclusions**: Although nursing students possess solid fundamental knowledge of exposure-related risks, a significant gap remains in their practical application and incident reporting. The high incidence of needlestick injuries (80.1%) underscores the importance of moving beyond theory toward enhanced clinical supervision. To address these gaps, nursing education should prioritize targeted practical training and cultivate a robust safety culture that encourages incident reporting.

## 1. Introduction

Occupational exposure to blood and body fluids presents a significant risk for healthcare workers [1], particularly nurses, who are frequently involved in patient care activities with potential for percutaneous or mucocutaneous injuries [2,3]. Such exposure incidents, including needlestick injuries, cuts from sharp instruments, and splashes of potentially infectious materials, remain a major source of transmission of blood-borne pathogens such as hepatitis B virus (HBV), hepatitis C virus (HCV), and Human Immunodeficiency Virus (HIV) [4,5,6]. The World Health Organization estimates that a substantial proportion of HBV and HCV infections among healthcare workers are attributable to occupational exposure incidents [7].

Nursing students, as future healthcare professionals, are particularly vulnerable due to limited clinical experience, frequent rotations in high-risk departments, and sometimes inadequate supervision during clinical practice [8,9,10,11]. Studies indicate that a significant proportion of nursing students experience at least one exposure incident during training, with needlestick injuries being the most common type [12,13,14,15]. Beyond the risk of infection, these incidents may provoke anxiety, psychological stress, and negatively impact students’ learning and attitudes toward clinical practice [16,17].

A high level of knowledge regarding exposure risks, standard precautions, immunization, and post-exposure management is essential for both prevention and appropriate response following an incident [18,19,20]. Guidelines from international and national health agencies emphasize structured educational interventions and regular training to improve awareness and preparedness among healthcare students and professionals [21,22,23]. Nevertheless, studies report varying levels of knowledge, suboptimal reporting of incidents, and persistent gaps in the implementation of standard precautions among nursing students in different settings [24,25]. Evidence from nursing student populations indicates that occupational safety knowledge, prior training, and safety-related practices vary substantially across educational settings [26].

Several factors may influence students’ knowledge and attitudes, including curriculum design, prior clinical exposure, participation in educational sessions, and institutional culture regarding occupational safety [27,28]. Given these influences, assessing nursing students’ baseline knowledge, experiences, and reporting behaviours related to occupational exposure is essential for identifying educational gaps and informing targeted interventions.

This study aimed to evaluate the level of knowledge, previous exposure experience, and reporting practices regarding occupational exposure incidents among nursing students at the Faculty of Health Sciences, University of Split, Croatia. Additionally, it aimed to identify demographic and educational factors associated with higher knowledge levels. By identifying specific knowledge gaps, particularly in post-exposure management, and examining educational and institutional factors associated with knowledge levels, this study provides context-specific insights into the Croatian nursing education setting. In Croatia, nursing education is delivered at both the secondary (vocational) and higher education levels, allowing students to enter undergraduate nursing programs from different secondary education backgrounds, which may influence their baseline knowledge and clinical preparedness [29]. Given the documented persistence of knowledge gaps across diverse healthcare education systems and the known underreporting of occupational exposure incidents among student populations, such findings may contribute to informing educational approaches and safety practices. Such an assessment is particularly timely in the Croatian context, where limited published data are available specifically on nursing students’ knowledge, exposure experiences, and reporting practices related to occupational incidents.

## 2. Materials and Methods

### 2.1. Study Design

This cross-sectional study was conducted among students enrolled at the Faculty of Health Sciences, University of Split, between May and June 2023. Inclusion criteria were active enrollment in the undergraduate nursing program and consent to participate. Students were recruited through classroom announcements, with voluntary participation and assurance of confidentiality. At the time of data collection, a total of 165 full-time students and 184 part-time students were enrolled in the undergraduate nursing program, representing a total of 349 eligible participants. In total, 274 students participated in the study, yielding a response rate of 78.5%.

### 2.2. Questionnaire

Data were collected using a structured, self-administered questionnaire specifically designed to assess knowledge related to occupational exposure incidents, their prevention, and post-exposure management, as well as students’ previous exposure experience, reporting behaviour, and perceived need for additional education. The questionnaire was newly developed in Croatian by the research team, based on relevant literature, national guidelines, and institutional protocols for safe handling of sharps implemented in Croatian healthcare settings [30,31,32]. Prior to data collection, the questionnaire was reviewed by the research team to assess the clarity, comprehensibility, and relevance of the items. The final instrument consisted of four sections covering: (1) demographic and academic characteristics (age, gender, year of study, student status (full-time or part-time), secondary school type and current nursing employment, (2) training and experiences practice (prior education on exposure incidents, personal experience of exposure, and incident reporting behaviors), (3) knowledge assessment (multiple-choice and true/false questions covering definitions, prevention, post-exposure procedures, immunization, and institutional policies related to occupational exposure risks institutional infection prevention and control (IPC) protocols), and (4) attitudes (items evaluating perceived need for further education on exposure risk and management) (see Appendix A for the full questionnaire).

The knowledge assessment section included 20 items, each scored as correct (1) or incorrect (0), yielding a total knowledge score for each participant ranging from 0 to 20, with higher scores indicating a higher level of knowledge. No missing responses were observed; therefore, no specific strategy for handling missing data was required. For statistical analyses, the total knowledge score (0–20) was treated as a continuous variable, and no categorical cut-off points for ‘low’, ‘moderate’ or ‘high’ knowledge were applied. Although the 20 knowledge items covered several content domains, they were summed into a single total knowledge score. Internal consistency of this 20-item knowledge scale, assessed using Cronbach’s alpha, was α = 0.49 in our sample, indicating low internal consistency, consistent with the heterogeneous content of the items.

For dissemination and publication purposes, this section of the administered questionnaire was professionally translated into English following rigorous forward and backward translation procedures to ensure conceptual and linguistic equivalence (Appendix A).

### 2.3. Statistical Analysis

Descriptive statistics were used to summarize participant characteristics, with categorical variables reported as frequencies and percentages. To examine factors associated with the total knowledge score on exposure incidents, prevention, and post-exposure management, both univariate and multivariable linear regression analyses were performed. In the univariate analysis, each independent variable was entered into the model separately. A multivariate linear regression analysis was conducted to adjust for potential confounding. The model included sociodemographic and educational variables such as age, gender, student status, year of study, type of secondary school, employment status, time since the last course on exposure incidents, prior experience with exposure incidents, and expressed additional education needs.

Results were reported as beta coefficients (β) with 95% confidence intervals (CI) and corresponding *p*-values. The reference categories were clearly defined for each categorical variable. Statistical significance was set at *p* < 0.050. All analyses were conducted using IBM SPSS Statistics for Windows (Version 21.0; IBM Corp., Armonk, NY, USA).

### 2.4. Ethical Approval

The study protocol was approved by the Ethics Committee of the Faculty of Health Sciences, University of Split (Approval number: 2181-228-103/1-31) on 28 April 2023. Prior to participation, all participants received detailed information about the aims, procedures, and voluntary nature of the participation. with informed consent obtained from each participant. All procedures were conducted in accordance with the Declaration of Helsinki.

## 3. Results

A total of 274 students participated in the study (response rate 78.51%), of whom 84.7% were female. The largest proportion of participants were aged 20–29 years (61%), were third-year students (38%), and had completed secondary education in nursing (75.9%). More than half of the respondents (64.6%) were currently employed in nursing, with the largest proportion of participants possessing 1–10 years of professional experience (38.1%) (Table 1).

Statistically significant differences were observed in age, gender, year of study, secondary education background, current nursing employment, and years of work experience (all *p* < 0.050). The distribution of student status (full-time vs. part-time) was comparable between groups (*p* = 0.809) (Table 1).

The proportion of students who had experienced an exposure incident and the timing since the last course attended differed significantly (both *p* < 0.001), whereas reporting rates and expressed need for additional education did not differ significantly between groups (Table 2).

One-third of participants (36.3%) reported having experienced an exposure incident. Of those, less than half (40.8%, n = 40) reported the incident to their mentor, instructor, or head nurse. Approximately one-third of respondents (34.4%) had attended a course on exposure incidents within the past six months, while nearly one-third (32.2%) had never attended such a course. Less than half of the participants (44.9%) expressed a need for further education on exposure incidents, prevention, and post-exposure measures (Table 2).

**Figure 1:**
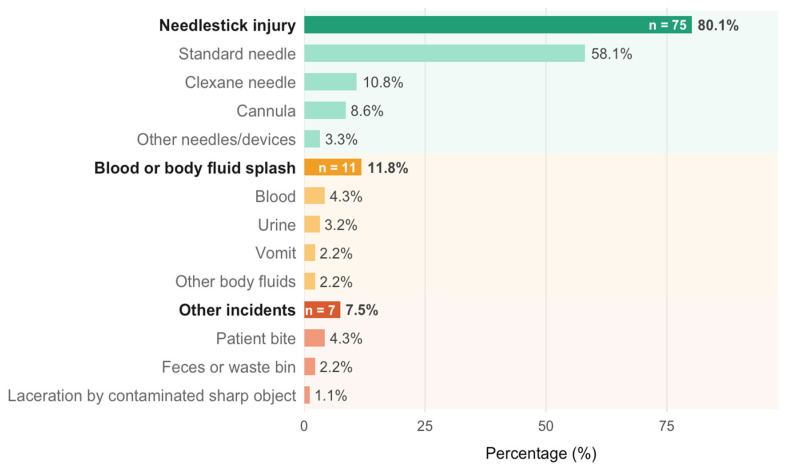
Types and subtypes of self-reported occupational exposure incidents among nursing students who experienced at least one incident (n = 93), expressed as percentages of all reported incidents.

Needlestick injuries accounted for the majority of exposure incidents (80.1%), most commonly involving standard needles (58.1%). Blood and body fluid splashes and other types of incidents were considerably less frequent (11.8% and 7.5%, respectively), highlighting needlestick injuries as the predominant occupational risk in this population (Figure 1).

**Figure 2:**
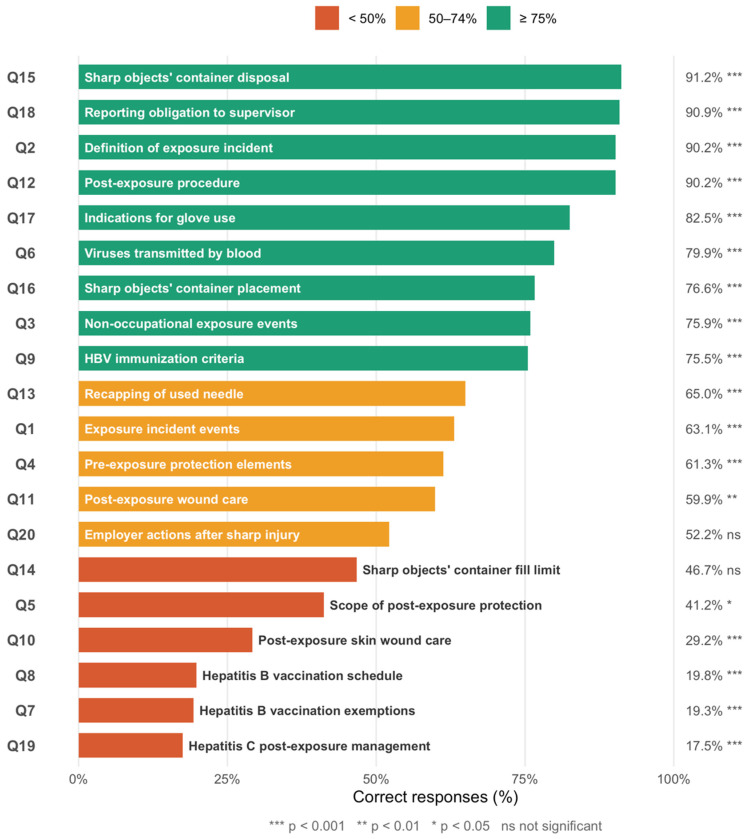
Item-level correct response rates for 20 knowledge questions on exposure incidents, prevention measures, and post-exposure management among nursing students (N = 274).

Figure 2 presents the correct response rates for all 20 knowledge items. Students performed best on items related to the disposal and placement of containers for sharp objects, the obligation to report exposure incidents, and the definition of professional exposure, all exceeding 90% correct responses. High accuracy was also observed for post-exposure procedures (90.9%) and indications for glove use (82.5%).

In contrast, marked knowledge gaps were identified for items concerning the hepatitis B vaccination schedule (19.8%), exemption groups (19.3%), hepatitis C post-exposure management (17.5%), and post-exposure skin wound care (29.2%). Statistically significant differences in correct response rates were observed for the majority of items (most *p* < 0.001), with the exception of required employer actions following a sharp object injury, with the exception of statutory employer responsibilities following percutaneous injuries (*p* = 0.468) and the sharp objects’ container fill limit (*p* = 0.277) (Figure 2).

The median total knowledge score was 12 (IQR: 11–14), with individual scores ranging from 3 to 19, indicating considerable variability in overall knowledge among students (Table 3).

Linear regression analysis examined factors associated with total knowledge scores on exposure incidents (Table 3). Univariate linear regression analysis provided an initial evaluation of which individual factors were associated with the total knowledge score. By examining each variable separately, two associations appeared noteworthy at the univariate level, such as full-time student status as a significant predictor of higher knowledge scores (β = 0.85. 95% CI: 0.24 to 1.46. *p* = 0.007), and first year of study that tended toward higher knowledge compared to third-year students, with borderline statistical significance (β = 0.64. 95% CI: −0.01 to 1.28. *p* = 0.051). No other variables were significantly associated with knowledge scores in univariate models (all *p* > 0.050) (Table 3).

In the multiple linear regression model, adjusting for all covariates, full-time student status remained a significant independent predictor of higher knowledge scores (β = 1.24. 95% CI: 0.29 to 2.18, *p* = 0.010). Additionally, first-year students had significantly higher scores compared to those in the third year (β = 0.82, 95% CI: 0.05 to 1.58, *p* = 0.036). No other characteristics were significantly associated with knowledge score after adjusting for other variables (all *p* > 0.050). The multivariate model reached statistical significance (*p* = 0.026), with an adjusted R^2^ of 4.6%. The Durbin–Watson statistic of 2.062 supported the assumption of independence of residuals, indicating no evidence of autocorrelation (Table 3).

## 4. Discussion

The present study provides a comprehensive assessment of knowledge, experiences, and reporting behaviors related to occupational exposure incidents among undergraduate nursing students at a single Croatian faculty. The findings reveal a notable discrepancy: while students demonstrate adequate understanding of fundamental safety principles, important gaps remain in post-exposure procedures, vaccination protocols, and wound care, areas where precise knowledge is important for preventing bloodborne infections.

These findings are particularly relevant in the European context. The Council of the European Union recommended in 2009 that patient safety principles be embedded in the education of all healthcare professionals; however, European nursing programmes continue to face challenges in implementing this recommendation. A recent mixed-methods study conducted across 16 faculties in the Czech Republic found that the dimensions most negatively rated by nursing students were “Frequency of events reported” (37.0%) and “Nonpunitive responses to errors” (42.4%), closely mirroring the underreporting patterns observed in the present study and suggesting that these challenges may not be limited to the Croatian context but may reflect a broader European phenomenon [33].

### 4.1. Knowledge Levels and Gaps

Our findings showed that undergraduate nursing students demonstrated generally adequate performance on items assessing fundamental safety measures and reporting procedures related to occupational exposure incidents, although these results should be interpreted with caution, given the low internal consistency of the knowledge scale (Cronbach’s α = 0.49), which limits its interpretation as a unified construct. A considerable variability in knowledge levels was observed, with individual scores ranging from 3 to 19 out of a possible 20. This wide range suggests that a subset of students may enter clinical practice with insufficient preparation, despite receiving the same formal curriculum. Students performed best on items assessing the disposal and placement of sharp objects’ containers, reporting obligations, and the definition of professional exposure; areas that are likely reinforced repeatedly throughout the nursing curriculum. In contrast, fewer than 30% of students correctly answered items on the hepatitis B vaccination schedule, hepatitis B vaccination exemption groups, hepatitis C post-exposure management, and post-exposure skin wound care. These findings highlight gaps in areas that are central to clinical decision-making in the immediate aftermath of an exposure incident, when correct and timely action is essential to minimize infection risk. Similar gaps have been reported by Datar et al., who found that healthcare students had insufficient knowledge of post-exposure prophylaxis and identified a gap between knowledge and actual preventive practice [34].

These findings are consistent with those of Al-Mugheed et al., who similarly identified gaps in knowledge of hepatitis B vaccination protocols and appropriate responses to potential hepatitis C exposure management among nursing students, despite adequate familiarity with infection-control principles [35]. The convergence of these findings across different healthcare systems and educational contexts suggests that these knowledge gaps may not be specific to a particular curriculum but may reflect broader challenges in how post-exposure management is taught and retained. Ledinski Fičko et al. and Al Qadire et al. similarly reported that nursing students across different settings demonstrate adequate baseline knowledge of needlestick injury recognition but consistently underperform on items requiring procedural specificity, especially regarding specific post-exposure actions, correct use of protective equipment, and understanding of institutional policies related to exposures [36,37].

Taken together, these findings suggest a gap between declarative knowledge, knowing that an incident must be managed, and procedural knowledge, knowing exactly how to manage it. Similar findings have been reported in previous studies, where nursing students demonstrated relatively high knowledge, attitude, and behavior scores, yet a substantial proportion still experienced needlestick and sharps injuries. This discrepancy between theoretical knowledge and actual clinical practice has also been observed in other settings, where unsafe practices such as needle recapping and low reporting rates persist despite adequate knowledge [38,39]. Similar patterns have also been observed among nurses, where no significant correlations were found between knowledge, attitudes, and practices, suggesting that knowledge alone may not be sufficient to ensure consistent adherence to safe clinical behavior [40]. Traditional didactic approaches may suffice for conveying factual information, but may be insufficient for developing the applied competencies required in the immediate aftermath of an exposure incident. A recent integrative review confirmed that nursing education still favours factual knowledge transmission over practical safety competence development, and that clinical education environments often fail to reinforce classroom-acquired knowledge [41]. Furthermore, Wu et al. demonstrated that virtual reality-based training using Gagne’s instructional model successfully reduced needlestick injury rates among nursing and medical interns, providing evidence that simulation-based and technology-enhanced pedagogical approaches can effectively bridge the declarative–procedural divide [42]. These findings suggest that curricula may benefit from greater emphasis on procedural competence, integrating case-based scenarios and simulation exercises that require students to apply specific post-exposure protocols under realistic conditions [43,44].

Notably, although nearly half of the respondents (44.9%) believed that they did not require additional knowledge regarding exposure incidents, our study revealed the opposite, highlighting a discrepancy between students’ perceived and actual knowledge levels. This overconfidence represents a potential safety concern, as overestimation of knowledge may reduce motivation to participate in additional training and hinder the adoption of updated guidelines and safe clinical practices, a pattern consistent with longitudinal evidence showing that first-year nursing students tend to be particularly overconfident about patient safety issues [45]. This discrepancy between perceived and actual competence can represent an underrecognized issue that may warrant further attention, including self-assessment exercises and objective structured evaluations that confront students with their own knowledge gaps [33,46].

### 4.2. Year of Study and Student Status as Factors Associated with Knowledge

Full-time students and first-year students demonstrated the highest knowledge scores regarding exposure incidents compared to students in the second and third years of study. The association with first-year status may reflect heightened vigilance and motivation upon entering clinical settings for the first time, where awareness of personal risk is acute and formal instruction on safety is most recent [37]. This finding may also be explained by the structure of the nursing curriculum. During the first year of study, within a foundational nursing course, “Nursing care”, students acquire basic knowledge and skills related to the prevention of needlestick injuries, management of exposure incidents, and the application of standard procedures and precautions. In later years, this topic is not systematically revisited within formal theoretical instruction. Instead, its reinforcement largely depends on clinical practice and mentorship, where variability in the consistency and emphasis of these topics may occur. On the other hand, third-year students showed lower knowledge regarding exposure incident prevention and management, a pattern consistent with longitudinal evidence suggesting that patient safety knowledge among nursing students tends to remain stable rather than accumulate over the course of training, while clinical competences may temporarily decline during the transition from classroom to clinical settings [45].

This may be of concern, given that third-year students are nearing the end of their training and will soon enter independent clinical practice, and may indicate the need for targeted refresher sessions on post-exposure procedures and reporting obligations in the final year of the program. It further suggests that knowledge acquired in early years is not being reinforced or updated and may be subject to decay as students become more accustomed to clinical environments and, paradoxically, less alert to risk, a finding that may warrant further attention from curriculum designers.

Al-Mugheed et al. reported the opposite trend, with senior students demonstrating greater knowledge than junior students [35], a finding that may reflect differences in curriculum structure, frequency of safety training, or the extent to which clinical supervisors actively reinforce safety protocols in the Saudi Arabian context. The contrasting direction of our findings underscores the importance of institutional and curricular context in shaping knowledge trajectories and suggests that exposure to clinical practice alone may not guarantee knowledge retention or improvement. This inconsistency across studies indicates that findings are not uniform and should be interpreted cautiously, taking into account contextual and methodological differences between study settings.

Full-time nursing students outperformed part-time students in multivariate analysis, consistent with findings by Cukljek et al., who observed that full-time students surpassed part-time peers in both knowledge and professional attitudes by the end of their education [47]. Part-time students, many of whom are already employed within the healthcare system, may rely more on experiential learning in clinical settings, which may not always align with current evidence-based guidelines [48]. This is particularly relevant given that prohibited sharps practices, such as needle recapping, may persist in clinical settings where experienced staff model unsafe behavior [49,50]. A concern explored further in Section 4.4.

Although the regression model explained a modest proportion of the variance in knowledge scores (adjusted R^2^ = 4.6%), this finding is consistent with previous research, where knowledge outcomes are influenced by multiple unmeasured factors. These may include mentorship quality, curriculum implementation, learning styles, and characteristics of the clinical environment. Other variables included in the multivariate model, although not statistically significant, may warrant further investigation in larger or more targeted samples. This includes time since the last course on exposure incidents, which was not significantly associated with knowledge scores in the present study. This finding may reflect variability in the content, quality, or retention of prior training, as previous research has shown that participation in safety training is associated with higher occupational health and safety scores among nursing students [26]. This suggests that temporal proximity to education alone may not be sufficient to ensure knowledge retention. Such modest explanatory power is not uncommon in studies of healthcare knowledge, where outcome variability is driven by numerous unmeasured factors, and should not diminish the clinical relevance of the identified predictors.

### 4.3. Exposure Incident Prevalence and Underreporting

More than one-third of participants (36.3%) reported having experienced an exposure incident during their training, a rate consistent with systematic reviews reporting prevalence estimates of 30–50% among nursing students globally [12]. Comparable findings were reported by Datar et al., who found that approximately one-quarter of healthcare students experienced needlestick or sharps injuries, with nursing students showing the highest prevalence [34]. This finding may be of concern. It means that a substantial proportion of nursing students are exposed to potential bloodborne pathogens before completing their education, during a period when they may lack both the knowledge and institutional support to respond appropriately.

The incidence of needlestick injuries in our study is consistent with rates reported among healthcare professionals globally [12,51]. For comparison, a study conducted in Saudi Arabia among nursing students reported a prevalence of only 14.1%, considerably lower than our findings, while only 22.6% of those injured reported the incident; fear and concern were cited as the main reasons for non-reporting. The lower prevalence reported in that study may reflect differences in clinical training intensity, supervision practices, or sample characteristics, but the similarly low reporting rates suggest that underreporting may be a cross-cultural phenomenon not specific to the Croatian context.

Another important finding is that fewer than half of those who experienced an incident (40.8%) reported the event. Comparable findings were reported in a recent study conducted among nursing students in Turkey, where 23.6% of participants experienced sharps-related injuries, and only 50% of these incidents were reported, further supporting the presence of underreporting in student populations [52]. Underreporting of occupational exposure incidents is a well-documented phenomenon across healthcare settings [49,51], but it is particularly problematic among students, who may fear academic consequences, feel uncertain about reporting procedures, or perceive the incident as insufficiently serious to warrant action. This is further supported by a narrative review by Hambridge, which highlighted that sharps injuries among healthcare students frequently occur across various clinical settings and are often underreported, with lack of knowledge identified as a contributing factor [53].

Alsabaani et al. similarly found that more than half of healthcare workers did not report needlestick injuries, even when they possessed adequate general knowledge of injury prevention [49]. The disconnect between knowledge of reporting obligations, which was among the highest-scoring items in our study (90.9% correct), and actual reporting behavior (40.8%) is striking and may suggest that barriers to reporting are not solely cognitive but also structural and cultural. Fear of stigma, lack of clear reporting pathways, time constraints during clinical rotations, and the perception that incidents will have no consequences if unreported are all potential contributing factors [12,54]. Indeed, Oweidat et al. found that the primary barriers to incident reporting among nurses were fear of disciplinary action and fear of being blamed, regardless of their level of awareness of reporting obligations [54]. Addressing underreporting, therefore, may require not only education but also the creation of a non-punitive safety culture in which students and nurses feel empowered and protected when disclosing incidents [54], a responsibility that may lie with both educational institutions and clinical placement sites. A comparable study conducted in Palestine found a non-reporting rate of 67.6% among nursing students who experienced needlestick injuries, with an overall exposure prevalence of 23.4% [55], further supporting the possibility that underreporting is a pervasive cross-cultural phenomenon. The consistent finding across diverse settings that students possessing adequate knowledge of reporting obligations still fail to report incidents may underscore the structural, rather than cognitive, nature of the barriers involved [46].

### 4.4. Needle Recapping and Safety-Engineered Devices

The most frequently reported needlestick injury among our participants involved a standard needle. A study conducted in Saudi Arabia among nursing students reported that needle recapping was again the primary cause of needlestick injuries, reported by 74.1% of participants [35]. Needle recapping was identified across multiple studies as the most common proximate cause of needlestick injuries [35,49,51]. This finding may be of concern, given that recapping has been explicitly prohibited by WHO for decades and is clearly outlined as an unacceptable practice in international IPC minimum standards [50]. The persistence of this practice in clinical settings, where nursing students observe and may imitate experienced staff, may highlight the importance of re-educating employed healthcare workers, not only students. A nursing student who has been taught correct technique in the classroom may quickly adopt the unsafe practices they observe during clinical placement if no corrective mechanism is in place; a phenomenon supported by evidence that inappropriate role modelling by clinical staff may contribute to professional identity dissonance in students and may undermine the safety behaviors instilled during formal training [33,56].

According to the latest WHO recommendations, recapping needles, bending needles, and manually removing needles from syringes are prohibited practices, clearly outlined in the minimum standards for Infection Prevention and Control (IPC) that all healthcare workers must adhere to during patient care [50]. The findings of the present study indicate the need for additional education on IPC minimum standards, as well as on the health and legal procedures available when an exposure incident is reported in a timely manner. Evidence from meta-analyses supports the use of safety-engineered devices (SEDs) in combination with education as the most effective strategy for reducing needlestick injuries, with greater reductions achieved than with education alone [57]. Therefore, in addition to education on exposure incidents, universities and healthcare institutions can benefit from ensuring the availability of safety-engineered consumable materials designed to prevent needlestick injuries. A recent decade-long retrospective analysis at a university teaching hospital demonstrated that sustained institutional commitment to education and safety training, including targeted programs for newly recruited staff, led to significant and progressive reductions in needlestick injury rates over time, providing further evidence that institutional-level educational interventions directed at both students and employed staff may be important for meaningful and sustained change [58].

### 4.5. Implications for Nursing Education and Policy

The findings of the present study have important implications for curriculum development and institutional policy. First, the identification of specific knowledge gaps, particularly in hepatitis B and C post-exposure management and wound care, should prompt a targeted review of how these topics are taught, assessed, and reinforced across all three years of the nursing program. Annual or biannual knowledge assessments on occupational safety topics can help identify students at risk of knowledge decay and trigger timely remedial education [45].

Second, the low reporting rate indicates the need for structured, mandatory training on incident reporting procedures, combined with institutional measures to reduce barriers and protect students who come forward. A recent study among nursing students also indicates that a considerable proportion experience sharps-related injuries, while reporting rates remain suboptimal, further supporting the need for structured education and continuous monitoring of adherence to safety protocols [52]. Practical measures that faculties and clinical partners can implement include: (a) anonymized digital reporting systems accessible via mobile devices, which reduce barriers related to fear of identification; (b) designated peer safety champions—fellow students trained to serve as first points of contact following an incident, providing immediate peer-level support; (c) mandatory structured debriefing sessions after each clinical rotation, during which students can discuss safety incidents in a supportive, non-judgmental environment; (d) explicitly stated non-punitive policies provided at the start of clinical rotations to encourage transparent incident dis-closure; (e) dedicated student safety officers at the faculty level who offer formal institutional guidance and support independently of clinical supervisors [54]. Evidence from multiple international settings supports the effectiveness of such approaches in improving incident reporting rates [33,59].

Third, the superior performance of full-time students suggests that the mode and intensity of academic engagement influences safety knowledge, independent of clinical experience. Part-time programs should explore whether supplementary safety modules or more frequent refresher sessions could close this gap [47,48].

Fourth, the present findings highlight the role of the clinical learning environment in shaping students’ occupational safety behaviors. This is supported by Kim and Park, who found that perceived safety of the clinical environment and positive attitudes toward infection control were significant predictors of compliance with infection prevention and control practices among graduating nursing students [60]. Clinical mentors and supervisors have a direct influence on students’ infection control practices, yet evidence suggests that mentors often struggle to assess the level of safety education their students have received prior to placement [61]. Healthcare institutions should invest in the regular professional development of clinical supervisors, explicitly addressing current IPC standards, prohibited sharps practices, and incident reporting obligations, ensuring that the clinical environment reinforces rather than contradicts what students are taught in the classroom [33,56].

### 4.6. Study Limitations

Several limitations of this study should be considered when interpreting these findings. The cross-sectional design prevents causal conclusions. Data were collected at a single faculty in Split, which may limit generalizability to nursing students in other Croatian institutions or other countries with different curricula and clinical training structures. The reliance on a self-administered questionnaire carries the risk of recall bias, particularly for items related to past exposure incidents and reporting behaviors, as well as social desirability bias in responses regarding adherence to safety protocols. In addition, the self-reported nature of the data may have contributed to underreporting of exposure incidents, particularly due to stigma, fear of negative consequences, or institutional culture. Furthermore, exposure experience and reporting behaviour were assessed using self-reported single items rather than validated multi-item scales. Therefore, these findings should be interpreted with caution, and the knowledge score should not be considered a direct proxy for actual clinical competence or safe behaviour. However, this was addressed by ensuring complete anonymity and the removal of all personally identifiable information. The multivariate model explained only 4.6% of the variance in knowledge scores, a finding consistent with previous research in nursing education, where knowledge scores are influenced by numerous latent variables, including individual motivation, cognitive learning styles, quality of clinical mentorship, and informal peer learning, that are difficult to capture through questionnaire-based assessments alone. Important factors associated with knowledge may therefore remain unidentified and should be explored in future research. In addition, although the questionnaire was developed based on relevant literature and guidelines, it was not formally subjected to pilot testing or comprehensive psychometric validation, and the internal consistency of the 20-item knowledge score was low (Cronbach’s alpha = 0.49). This indicates that the instrument should not be regarded as a homogeneous psychometric scale, and the knowledge score should be interpreted with caution and not as a direct proxy for actual clinical competence or safe behaviour.

## 5. Conclusions

This study indicates that nursing students demonstrate generally adequate performance on knowledge items related to occupational safety, particularly regarding the prevention and reporting of occupational exposure incidents. However, given the low internal consistency of the knowledge instrument (Cronbach’s α = 0.49), these findings should be interpreted with caution and not taken as evidence of a well-defined, unified knowledge construct. Also, a considerable variability in knowledge levels was observed, along with significant gaps in areas such as hepatitis B vaccination, post-exposure management of hepatitis C, and appropriate wound care. Importantly, the discrepancy between students’ high self-assessed knowledge and their actual performance on specific post-exposure items suggests that overconfidence may represent an underappreciated barrier to continued learning in this area.

More than one-third of students experienced an exposure incident, while fewer than half of these incidents were reported, suggesting the presence of barriers within the reporting system and a possible underestimation of exposure-related risks. Needle stick injuries remain the most common form of occupational exposure, with standard needle injuries predominating and needle recapping identified as a key contributing factor despite being explicitly prohibited. Given that the gap between knowledge of reporting obligations (90.9% correct) and actual reporting behavior (40.8%) cannot be attributed solely to cognitive deficits, institutional and cultural interventions, including non-punitive reporting policies and anonymized reporting systems, may be considered in this context.

Full-time and first-year nursing students demonstrated higher levels of knowledge, suggesting the importance of continuous education and regular reinforcement of both theoretical and practical content. The findings suggest that more structured educational approaches, increased emphasis on practical training, and a stronger safety culture may be beneficial within similar educational settings. Future research should employ longitudinal designs to track knowledge trajectories across all years of study and evaluate the effectiveness of specific educational interventions, including simulation-based training and virtual reality approaches, in improving both knowledge retention and reporting behavior. However, given the single-center, cross-sectional design and the limited explanatory power of the model, these findings should be interpreted as context-specific and exploratory rather than generalizable to broader educational systems.

## Figures and Tables

**Table 1:** Demographic characteristics of respondents (N = 274).

	n (%)	*p*-Value *
**Age**		
18–19	8 (2.9)	<0.001
20–29	167 (61.0)	
30–39	65 (23.7)	
40–49	28 (10.2)	
50–59	6 (2.2)	
**Gender**		
Male	42 (15.3)	<0.001
Female	232 (84.7)	
**Student status**		
Full-time	135 (49.3)	0.809
Part-time	139 (50.7)	
**Year of Study**		
First	99 (36.1)	0.031
Second	71 (25.9)	
Third	104 (38.0)	
**Secondary school type**		
Medical Secondary School (nurse/technician)	208 (75.9)	<0.001
Medical Secondary School (other)	18 (6.6)	
Grammar School	46 (16.8)	
Other four-year secondary school	2 (0.7)	
**Current nursing employment**		
Yes	177 (64.6)	<0.001
No	96 (35.0)	
**Years of clinical experience**		
none	90 (33.0)	<0.001
<1	7 (2.6)	
1–10	104 (38.1)	
11–20	52 (19.0)	
21–30	17 (6.2)	
31–40	3 (1.1)	

* *p*-values were calculated using the chi-square test.

**Table 2:** Exposure incident experience, reporting behavior, course attendance, and educational needs among nursing students (N = 274).

	n (%)	*p*-Value *
**Time since last course on exposure incidents**		
Less than 6 months	92 (34.4)	
6 months to 1 year	60 (22.2)	
1 to 2 years	18 (6.7)	<0.001
More than 2 years	12 (4.4)	
I have not attended such a course so far	87 (32.2)	
**Experienced exposure incident**		
Yes	98 (36.3)	<0.001
No	172 (63.7)	
**Reported exposure incident †**		
Yes	40 (40.8)	0.069
No	58 (59.2)	
**Expressed need for education on exposure incidents, prevention, and post-exposure measures**		
Yes	123 (44.9)	0.091
No	151 (55.1)	

* *p*-values were calculated using the chi-square test. † Calculated only among students who reported experiencing an exposure incident (n = 98).

**Table 3:** Characteristics associated with total knowledge score on occupational exposure incidents, prevention, and post-exposure management among nursing students (N = 274), determined by univariate and multivariable linear regression.

	Univariate Beta (95% CI); *p*-Value	Multivariate Beta (95% CI); *p*-Value
**Age** (years)	−0.03 (−0.07, 0.01); 0.125	0.01 (−0.04, 0.06); 0.709
**Gender** (Ref. female)		
Male	−0.50 (−1.36, 0.36); 0.250	−0.28 (−1.17, 0.62); 0.545
**Student status** (Ref. part-time)		
Full-time	0.85 (0.24, 1.46); 0.007	1.24 (0.29, 2.18); 0.010
**Year of study** (Ref. 3rd)		
1st	0.64 (−0.01, 1.28); 0.051	0.82 (0.05, 1.58); 0.036
2nd	0.50 (−0.21, 1.20); 0.166	0.58 (−0.26, 1.41); 0.175
**Finished secondary school** (Ref. Medical Secondary School (nurse/technician))		
Medical Secondary School (other)	−0.42 (−1.66, 0.83); 0.507	−0.75 (−2.1, 0.61); 0.279
Grammar School	0.80 (−0.75, 0.91); 0.848	−0.58 (−1.61, 0.45); 0.267
Other four-year secondary school	0.22 (−3.41, 3.85); 0.905	0.55 (−3.21, 4.30); 0.774
**Current nursing employment** (Ref. No)		
Yes	−0.62 (−1.30, 0.02); 0.059	−0.06 (−1.08, 0.96); 0.91
**Time since last course on exposure incidents** (Ref. I have not attended such a course so far)		
Less than 6 months	0.02 (−0.64, 0.67); 0.955	0.02 (−0.76, 0.8); 0.958
6 months to 1 year	0.66 (−0.08, 1.41); 0.079	0.48 (−0.39, 1.34); 0.279
1 to 2 years	−0.84 (−2.08, 0.41); 0.186	−0.74 (−2.07, 0.59); 0.273
More than 2 years	−0.82 (−2.32, 0.69); 0.286	−0.93 (−2.57, 0.71); 0.264
**Experienced an exposure incident** (Ref. No)		
Yes	0.10 (−0.54, 0.75); 0.754	−0.01 (−0.67, 0.66); 0.981
**Attitudes toward the need for education on exposure incidents, prevention, and post-exposure measures** (Ref. No)		
Yes	−0.48 (−1.10, 0.14); 0.128	0.64 (−0.04, 1.31); 0.064

95% confidence intervals = 95% CI. Ref. = reference category.

## Data Availability

The data presented in this study are available upon reasonable request from the corresponding author.

## Public Involvement Statement

No public involvement in any aspect of this research.

## Guidelines and Standards Statement

This manuscript was prepared in accordance with the STROBE guidelines for reporting observational studies.

## Abbreviations

The following abbreviations are used in this manuscript:

HBV	Hepatitis B virus
HCV	Hepatitis C virus
HIV	Human Immunodeficiency Virus
IPC	Infection Prevention and Control
WHO	World Health Organization

## Appendix A

This appendix presents the English translation of the questionnaire on exposure incidents, prevention, and post-exposure management, developed in accordance with relevant guidelines [30,31,32]. Correct answers are indicated in bold.

Q1. The term “exposure incident” includes:

1. A needlestick or cut with a sharp object contaminated with blood or another body fluid or tissue of the patient.
2. Contact of mucous membranes (eyes, nose, mouth, or genitals) or damaged skin with blood or another body fluid/tissue of the patient (e.g., blood splashing into the eyes or unprotected sexual intercourse).
3. A human bite that breaks the skin.
4. **All of the above are correct.**
5. All are correct except Answer 2.

Q2. Occupational exposure (exposure incident) is contact by a healthcare or non-healthcare worker with the blood or body fluids and tissues of a patient that may contain blood-borne pathogens and can cause infection, occurring during the performance of work or professional activities.

1. **True**
2. False

Q3. Non-occupational exposure includes:

1. Needlestick injury with a needle contaminated with the patient’s blood
2. **Unprotected risky sexual intercourse**
3. Cut with a sharp object contaminated with the patient’s blood

Q4. Pre-exposure protection includes all of the following except:

1. Education on protective measures and equipment
2. Hepatitis B vaccination
3. Use of protective equipment (gloves and mask)
4. **Short-term use of medications (vaccines, immunoglobulins, antimicrobial drugs)**

Q5. Post-exposure protection encompasses a set of activities aimed at reducing the risk of blood-borne infection in an injured healthcare or non-healthcare worker before the exposure incident has occurred.

1. True
2. **False**

Q6. The most common blood-borne viruses include:

1. **Hepatitis B. Hepatitis C. Human immunodeficiency virus (HIV)**
2. Hepatitis C. Hepatitis A. HIV
3. Hepatitis A. Herpes Zoster

Q7. Hepatitis B vaccination is compulsory for all the following groups except:

1. Patients on hemodialysis
2. Persons in healthcare and other institutions, as well as healthcare workers in private practice
3. Sexual partners and household contacts of HBsAg-positive individuals
4. Employees providing accommodation services for people with mental and intellectual disabilities
5. Intravenous drug users
6. Patients with hemophilia and leukemia
7. **Newborns of HBsAg-negative mothers**

Q8. Hepatitis B vaccination is administered according to the 0, 3, and 6-month schedule.

1. True
2. **False**

Q9. A person is considered protected (“vaccinated”) against HBV infection if they have received all three prescribed doses of the HBV vaccine, and one month after the last dose. developed a protective titer of anti-HBs antibodies >10 mIU/mL.

1. **True**
2. False

Q10. After exposure, in the case of a needlestick, cut or other skin injury with a sharp object, the injured skin should be washed with soap and water.

1. True
2. **False**

Q11. After exposure, the injured site should be allowed to bleed freely and then covered lightly.

1. **True**
2. False

Q12. After exposure, mucous membranes, eyes, and mouth should be flushed abundantly with saline or water.

1. **True**
2. False

Q13. A cap should be placed back on a used needle.

1. True
2. **False**

Q14. A sharps container may be filled:

1. Up to half of its volume
2. **Up to two-thirds of its volume**
3. Up to one quarter of its volume
4. Completely

Q15. A sharps container is disposed of as:

1. Municipal waste
2. **Infectious waste**

Q16. A sharps container does not necessarily need to be in the immediate work area (e.g., when taking blood samples or administering therapy).

1. True
2. **False**

Q17. Gloves are used when contact with blood, body fluids, and contaminated objects is likely.

1. **True**
2. False

Q18. A person who has had an incident must report it to their immediate supervisor.

1. **True**
2. False

Q19. Post-exposure procedure for hepatitis C includes the use of post-exposure prophylaxis (PEP).

1. True
2. **False**

Q20. In case of a worker’s injury with a sharp object, the employer is required to:

1. Immediately implement the prescribed standard operating procedures
2. Ensure healthcare for the injured worker
3. Determine the causes and circumstances of the injury and record all exposure incidents
4. Send a report of each sharps injury to the Croatian Institute of Public Health (HZJZ)
5. **All of the above are correct**
6. All are correct except for Answer 4
